# Not Every Dip Matters: Cerebral Oxygenation Variability and Behavioral Outcomes in Children

**DOI:** 10.1111/aas.70147

**Published:** 2025-11-06

**Authors:** Jacob Karlsson, Laszlo Vutskits

**Affiliations:** ^1^ Pediatric Perioperative Medicine and Intensive Care Karolinska University Hospital Stockholm Sweden; ^2^ Department of Physiology and Pharmacology (FYFA) Karolinska Institute, C3, Per‐Arne Lönnqvist Group ‐ Section of Anesthesiology and Intensive Care, Anestesi‐ och Intensivvårdsavdelningen Stockholm Sweden; ^3^ Department of Anesthesiology, Pharmacology, Intensive Care and Emergency Medicine University Hospital of Geneva Geneva Switzerland; ^4^ Geneva Neuroscience Center University of Geneva Geneva Switzerland

Near‐infrared spectroscopy (NIRS) has become an increasingly popular tool to non‐invasively monitor cerebral oxygenation in children under anesthesia. Its appeal is obvious: a simple measurement, obtained from a sticky probe on the forehead gives monitoring access to the brain and a numerical measure of cerebral saturation. This number can then be used to optimize oxygenation of the anesthetized brain. Even if NIRS only monitors a small part of the brain (a unilateral probe on the forehead only reflects a few percent of the whole brain volume at the most), this can still be tremendously helpful to guide intraoperative management. This is the case, for example, in pediatric cardiac surgery where intraoperative NIRS monitoring during aortic cannula placement and while on cardiopulmonary bypass can be used to adjust the surgical technique and/or optimize the parameters of extracorporeal circulation.

Interestingly, in the broader context of general pediatric anesthesia, where postoperative behavioral effects of hypnotic agents have been a subject of debate for the last decade, NIRS has been used in hope of linking a numerical value to meaningful postoperative cognitive outcomes. Such an assumption is fuelled by observational, and mostly retrospective, studies in the domain of congenital cardiac surgery suggesting an association between lower perioperative NIRS values and impaired neurodevelopmental outcome in young children [1, 2, 3]. Yet, as with many emerging monitoring modalities, there is a risk for over‐enthusiastic interpretation of the obtained numbers, often overlooking the clinical context or the method's limitations.

A recent study by Gómez‐Pesquera et al. reported a surprising association between even minimal cerebral desaturations—as little as 5 percentage points below baseline—and negative postoperative behavioral changes (NPOBC) in children [4]. If confirmed, these results would indicate that even tiny fluctuations may be associated with unwanted short‐ and long‐term consequences. Against the background of that study, two questions arise: first, are small variations truly dangerous, or simply part of normal physiology? Second, how small a change in cerebral saturation (rSO_2_) can a NIRS probe actually detect?

In this issue of *Acta Anaesthesiologica Scandinavica*, Kälvesten et al. revisit Gómez‐Pesquera's hypothesis on cerebral desaturation and NPOBC in a prospective cohort of 180 healthy children aged 2–6 years undergoing routine non‐cardiac surgery [5]. The main outcome was the occurrence of parentally graded NPOBC after 7 days in relation to exposure to 5, 10, 15, or 20 percentage point dips in rSO_2_ from baseline, sustained for > 2 min. Their findings are both reassuring and cautionary: Cerebral oxygenation remained stable under anesthesia, with reductions ≥ 5 percentage points occurring in only 3% of cases and no desaturations ≥ 15 percentage points. No association between intraoperative cerebral desaturation and NPOBC could be demonstrated.

As mentioned above, the results from Gómez's study as well as Kälvesten's study invite a broader discussion on what really constitutes a meaningful change.

Physiological systems are associated with inherent variability, and small fluctuations in oxygenation are a part of daily life—from sleeping to running, to children flying in a pressurized cabin thousands of meters above sea level. Most everyday variations are well tolerated thanks to compensatory mechanisms, including adjustments in cerebral blood flow and oxygen extraction.

In a study on the physiological effects of staying at high‐altitude mountain resorts, children 3 to 36 months exposed to 3109 m, sustained for 24 h, showed a decrease in cerebral tissue oxygenation measured by NIRS typically by around 10–12 percentage points compared to ground values. Younger infants showed the largest reductions—yet without evidence of intracranial hypertension or neurological impairment [6]. In fact, thousands of children flying commercially every day are subjected to similar scenarios and it is unlikely that this is linked to negative behavior (apart from frustrating jet lag). Similarly, critically ill children transported by air show transient decreases in cerebral oxygen saturation at altitude [7]. These examples from flying and high altitude demonstrate that mild cerebral desaturations are relatively common and not always harmful. Naturally, the situation under anesthesia is somewhat different, since anesthesia in itself may affect some of these compensatory mechanisms designed to maintain oxygen delivery. Nevertheless, small deviations in oxygenation are unlikely to result in measurable postoperative outcomes.

In addition to the magnitude of change, the duration of exposure is equally important. Using a 2‐min cut‐off in Kälvesten's study thus makes sense rather than focusing on short dips. Considering this, area‐under‐the‐curve metrics (much like radiation exposure) may be more informative than focusing on how many percentage points rSO_2_ drops. Future research should therefore include data on deeper or sustained desaturations, cumulative burden, and the interaction of oxygenation changes with other physiological variables known to influence cerebral oxygenation, such as blood pressure, cardiac output, and end‐tidal CO_2_. In this context, it is important to note that, although no study has yet examined whether maintaining cerebral NIRS within a predefined range influences outcomes in pediatric perioperative care, evidence from the neonatal literature unfortunately suggests that focusing on NIRS alone may not improve outcomes. Indeed, while two randomized controlled trials in extremely preterm newborns revealed important differences in cerebral NIRS values between patients where this parameter was maintained in a defined “physiological range”, with dedicated treatment guidelines versus those where clinicians were blinded to this monitoring modality, no differences in mortality and major morbidities, including brain injury could be identified between the two groups [8, 9]. In light of these results, and despite the obvious differences between extreme preterm newborns necessitating intensive care and otherwise healthy children undergoing routine general anesthesia, it is difficult to imagine that relatively minor changes in NIRS values may show an association with relatively subtle neurobehavioral problems.

The study by Kälvesten et al. also raises another important methodological question to address. Definitions of cerebral desaturation vary widely between studies. In addition, there is often confusion about whether relative percentage drops or actual changes in percentage points are used when defining the desaturations. Just as important as deciding what constitutes a physiologically meaningful change, is defining the minimal change the measuring device can measure. Of particular importance in this context is the reporting standard of a monitoring device's inherent precision—in other words, what is the minimal change a monitor can detect as a real change and not just an effect of inherent measurement error of the method. Inherent precision should therefore always be reported as soon as a monitoring device is used to track changes [10]. It is also important to ensure that the recorded changes are greater than the least significant change detectable by the device.

Even considering the challenges outlined above, Kälvesten's study still offers a reassuring message: in the context of anesthesia for routine pediatric surgery, significant cerebral desaturations are uncommon, and small fluctuations do not predict postoperative behavioral changes.

As our understanding of cerebral oxygen dynamics deepens, it is worth remembering that variability is generally a sign of normal physiology, not necessarily a marker of pathology. The developing brain may be fragile—but it is also an adaptable organ designed to function across a spectrum of oxygenation states, whether in the operating room, at altitude, or somewhere in between. Measuring variability is important but such changes need to be clearly understood within the clinical context. In other words, not every dip in rSO_2_ in NIRS matters.

## Author Contributions

J.K. and L.V. contributed equally to idea, draft and completion of the manuscript.

## Conflicts of Interest

The authors declare no conflicts of interest.

## Data Availability

Data sharing not applicable to this article as no datasets were generated or analyzed during the current study.
